# Hematologic Inflammation Indices for Differentiating between Brucella, Pyogenic, and Tuberculous Spondylodiscitis

**DOI:** 10.3390/biomedicines12092059

**Published:** 2024-09-10

**Authors:** Ali Irfan Baran, Irfan Binici, Yusuf Arslan, Zekiye Hakseven Karaduman, Server Ilter, Tayyar Tarcan, Murat Unal

**Affiliations:** 1 Department of Infectious Diseases and Clinical Microbiology, Faculty of Medicine, Van Yuzuncu Yıl University, Van 65080, Turkey; 2 Department of Infectious Diseases and Clinical Microbiology, Batman Training and Research Hospital, Batman 72000, Turkey; 3 Department of Infectious Diseases and Clinical Microbiology, Van Training and Research Hospital, Van 65300, Turkey; 4 Department of Physical Therapy and Rehabilitation, Faculty of Medicine, Van Yuzuncu Yıl University, Van 65090, Turkey; 5 Department of Infectious Diseases and Clinical Microbiology, Tatvan State Hosptial, Bitlis 13000, Turkey; 6 Department of Infectious Diseases and Clinical Microbiology, Nusaybin State Hosptial, Mardin 47300, Turkey

**Keywords:** neutrophil-to-lymphocyte ratio, spondylodiscitis, inflammation, infection, brucellosis, pyogenic, tuberculous

## Abstract

Infectious spondylodiscitis is a life-threatening disease and has some challenges in terms of diagnostic, differentiative, and therapeutic processes. Therefore, rapid and effective management of infectious spondylodiscitis is necessary. Hematological inflammation indices (HIIs) such as the neutrophil/lymphocyte ratio and aggregate index of systemic inflammation are derived from blood cells and used as diagnostic, prognostic, predictive, and treatment monitoring indicators. This study aimed to evaluate HIIs for discriminating between infectious spondylodiscitis pathogens. This retrospective comparative study included 116 patients with infectious spondylodiscitis. According to the responsible infectious pathogens, three types of infectious spondylodiscitis were defined: *Brucella* (n = 51), pyogenic (n = 43), and tuberculous (n = 22). The HIIs were derived from baseline complete blood counts. The three types of infectious spondylodiscitis were statistically compared for the HII scores. We found that the *Brucella* group had significantly lower HII scores than the pyogenic group (*p* < 0.05). Also, the *Brucella* group had significantly lower HII scores than the tuberculous group (*p* < 0.05). However, no significant differences were found between the pyogenic and tuberculous groups regarding HIIs (*p* > 0.05). In conclusion, the HIIs may be considered in the differentiation between *Brucella* spondylodiscitis and other types of infectious spondylodiscitis.

## 1. Introduction 

Infectious spondylodiscitis is a severe form of spinal infection. Its incidence has increased in the modern era due to increased rates of individuals with various risk factors such as older age, chronic illness, comorbidities, immunodeficiency, steroid use, drug abuse, spinal interventions, and surgeries [1,2]. Infectious spondylodiscitis is associated with non-specific clinical features such as back pain, limited mobility, and high fever [3,4]. Since infectious spondylodiscitis is rare and low back pain is very common in society, diagnosis may be missed or delayed [4,5,6,7]. The diagnosis of infectious spondylodiscitis is based on a careful history, clinical symptoms, physical examination, serological and hematological data, radiological findings, microbiological culture, histological and molecular analyses, and tissue samples [6,7,8]. Due to challenges in the diagnosis, differential diagnosis, and treatment processes, unsuccessful treatment outcomes and severe complications requiring surgical intervention may develop [3,5,6,8]. Surgical intervention is recommended in patients with refractory symptoms, unstable spine, abscess, and neurologic deficit; conservative and multidisciplinary management including multiple antibiotics, spine bracing, and bed rest is needed for all patients with infectious spondylodiscitis [4,9,10]. However, considering the seriousness of the disease, it is urgently necessary to detect the responsible microorganisms in order to select the most suitable treatment as soon as possible [6,7,8]. Unfortunately, non-specific symptoms and findings and time-consuming, advanced, and expensive diagnostic tests not available in every center make rapid intervention impossible. Therefore, there is a need for a more appropriate diagnostic test, and it is clear that such a test must be fast, simple, easy, accessible, and inexpensive. Thanks to such a test, rapid management can be provided, including early diagnosis, differential diagnosis, and treatment procedures.

*Brucella* spondylodiscitis is one of the most common forms of osteoarticular brucellosis and also of spinal brucellosis [11]. Osteoarticular brucellosis has been reported to be associated with delayed diagnosis and prolonged disease duration before diagnosis [12]. Also, *Brucella* spondylodiscitis is associated with neurological deficits and abscess formations in various tissues [11].

Pyogenic spondylodiscitis is the most common subtype of infectious spondylodiscitis [13]. A meta-analysis has shown that early surgery in pyogenic spondylodiscitis is superior to non-surgical interventions in terms of the risk of treatment failure and morbidity and mortality [13].

Tuberculous spondylodiscitis (Pott’s disease) is a severe manifestation of musculoskeletal tuberculosis and one of the most common sites of musculoskeletal involvement [14,15]. Because of its severely damaging nature and disabling complications, tuberculous spondylodiscitis should be diagnosed rapidly and treated promptly [15].

It is well known that rapid management of these clinical conditions, including early diagnosis, differentiation, and therapeutic interventions, is of paramount importance and can be of paramount benefit. These three diseases have the potential for significant health and economic costs in the event of delay, particularly in endemic regions. Although efforts are being made to develop more effective applications for the management of these diseases [13,16,17,18,19], the need for faster and easier diagnosis, differentiation, and therapeutic interventions remains.

Currently, hematological inflammatory indices (HIIs) are popular indicators. They are derived simply from complete blood count and therefore are speedy, reliable, effortless, objective, reproducible, and cost-effective indices. In addition to blood cells, the commonly suggested and used HIIs are NLR (neutrophil/lymphocyte), MLR (monocyte/lymphocyte), PLR (platelet/lymphocyte), NLPR [neutrophil/(lymphocyte × platelet)], SII (systemic immune/inflammation index, neutrophil × platelet/lymphocyte), SIRI (systemic inflammatory response index, neutrophil × monocyte/lymphocyte), and AISI (aggregate index of systemic inflammation, neutrophil × platelet × monocyte/lymphocyte) (Table 1) [20,21,22]. These HIIs are recommended as diagnostic [23,24], prognostic [21,22,23,24,25], predictive [22,26], and treatment response [27] markers in various medical conditions such as idiopathic pulmonary fibrosis [21], COVID-19 [22], glaucoma [23], Cushing’s disease [24], sepsis [25], gastric cancer [26], and psoriasis [27]. In addition, HIIs have been shown to be helpful in diagnosing infectious diseases such as acute appendicitis and determining the presence of complications [28]. Moreover, an exceptional contribution of HIIs to diagnosis as a part of diagnostic scores in acute appendicitis has been revealed [29]. These indices may have the ability to discriminate between infectious and non-infectious diseases, as well as between different infectious diseases. Therefore, the diagnostic and differential role of HII in clinical infectious diseases such as infectious spondylodiscitis seems to be a topic worth investigating.

In addition, it makes sense that researchers looking for a test for early diagnosis and differential diagnosis should focus on changes and features in the early stages of the disease that have not yet been affected by factors such as treatment.

In this study, we hypothesized that baseline (pre-treatment) HIIs may be useful indicators in the differentiation between microbiological pathogens responsible for infectious spondylodiscitis. Therefore, we aimed to compare baseline (pre-treatment) HII scores between *Brucella*, pyogenic, and tuberculous spondylodiscitis. If the hypothesis is confirmed, HIIs can be used in every center to predict the responsible infectious agent. In this way, faster and more successful diagnostic, differentiative, and therapeutic processes can be provided.

## 2. Materials and Methods

### 2.1. Design of the Study

This was a retrospective comparative study assessing patients with infectious spondylodiscitis in terms of HIIs derived from baseline complete blood counts. Patients’ data from 2014 to 2023 were obtained from our Hospital Information Systems. The hospital’s Ethics Committee approved the study design (reference number 2024/3-32, date of approval 8 March 2024). The study was carried out in accordance with the principles of the Declaration of Helsinki. 

Patients’ demographics and clinical, radiological, and laboratory characteristics were recorded. The HIIs (NLR, MLR, PLR, NLPR, SII, SIRI, and AISI) were derived from baseline complete blood counts.

In total, 116 patients diagnosed with infectious spondylodiscitis were included in this study. Based on the types of infectious agents, *Brucella* (n = 51), pyogenic (n = 43), and tuberculous (n = 22) spondylodiscitis was diagnosed.

### 2.2. Diagnosis of Brucella Spondylodiscitis 

Alongside symptoms and findings, *Brucella* spondylodiscitis was diagnosed if the following findings were present: magnetic resonance imaging (MRI) showing spondylodiscitis plus (≥1/160 serum *Brucella* tube agglutination titer or brucella positive blood culture).

### 2.3. Diagnosis of Pyogenic Spondylodiscitis 

In addition to complaints and findings, the diagnosis of pyogenic spondylodiscitis was considered if the following findings were observed: MRI showing spondylodiscitis plus pyogenic bacteria detection in the samples taken by radiological or surgical interventions and response to antibiotherapy for pyogenic bacteria.

### 2.4. Diagnosis of Tuberculous Spondylodiscitis 

Patients with MRI showing spondylodiscitis were diagnosed as tuberculous spondylodiscitis when acid-fast bacilli (Ziehl–Neelsen stain) and/or polymerase chain reaction and/or culture positive for *Mycobacterium tuberculosis* and/or caseating/chronic granulomatous samples were obtained by radiological or surgical interventions. Also, clinical, radiological, and laboratory results indicating tuberculosis plus positive quantiferon and/or tuberculin skin test, and response to antituberculosis treatment, were used to diagnose tuberculous spondylodiscitis.

### 2.5. Inclusion Criteria 

Inclusion criteria were as follows: a diagnosis of brucella, pyogenic, or tuberculous spondylodiscitis in the past 10 years, presence of lumbar MRI and baseline complete blood count, lumbar spine involvements, and age ≥ 18 years.

### 2.6. Exclusion Criteria

Exclusion criteria were as follows: patients without adequate data, age < 18 years, combination of infectious spondylodiscitis types, other pathogens of infectious spondylodiscitis such as fungal spinal infection, non-infectious forms of spondylodiscitis such as Modic changes and ankylosing spondylitis, recurrent infectious spondylodiscitis, and spine involvements outside the lumbar region. 

The groups were statistically evaluated and compared for baseline features such as age, gender, symptom duration, C-reactive protein (CRP), erythrocyte sedimentation rate (ESR), complete blood count, and complete blood count-derived HII.

### 2.7. Statistical Analyses

All data were analyzed using IBM SPSS version 20.0 (IBM Corp., Armonk, NY, USA). The Kolmogorov–Smirnov test was applied to evaluate if continuous variables had a normal distribution. The abnormally distributed variables were the SIRI and AISI in the *Brucella* group (BG), the NLR, MLR, and AISI in the pyogenic group (PG), and the SIRI in the tuberculous group (TG). Accordingly, for the abnormally distributed variables, the Mann–Whitney U test was used in the comparisons between the groups. For the variables with normal distribution, parametric (One-Way ANOVA Bonferroni post hoc test, and Independent *t*-test) were used in the comparative analyses. Fisher’s exact test was used in the comparisons of categorical variables. Statistical significance was set at *p* ≤ 0.05. 

## 3. Results

The BG, PG, and TG were similar in terms of age and gender (*p* > 0.05). Considering symptom duration, there was a similarity between the BG and PG (*p* = 0.804), while the TG had a higher score than the other groups (*p* < 0.001). Considering CRP and ESR levels, the BG and PG were similar to the TG, while the BG had significantly lower scores for CRP (*p* < 0.021) and ESR (*p* < 0.019) (Table 2).

In terms of blood cells, patients in the BG had significantly lower leukocyte and neutrophil counts than both the PG (*p* < 0.001 and *p* < 0.001) and the TG (*p* = 0.044 and *p* = 0.019). However, PG and TG were statistically similar for leukocyte (*p* = 0.147) and neutrophil (*p* = 0.139) counts. In addition, the three groups were statistically similar for monocyte and platelet counts (*p* > 0.05) (Table 3).

Considering HIIs, the BG had lower scores than the PG in the investigated indices (NLR, MLR, PLR, NLPR, SII, SIRI, and AISI) (*p* < 0.05). Also, the BG had lower scores than the TG in the NLR, MLR, SII, SIRI, and AISI (*p* < 0.05), but these two groups were similar for the PLR and NLPR (*p* > 0.05). No significant difference was found between the PG and TG for all the HIIs (*p* > 0.05) (Table 4).

## 4. Discussion

This comparative study focused on the differences between brucella, pyogenic, and tuberculous spondylodiscitis in terms of baseline (pre-treatment) complete blood count and, especially, complete blood count-derived HIIs. The results revealed that the HIIs (NLR, MLR, PLR, NLPR, SII, SIRI, and AISI) were significantly lower in the BG than those in the PG. Also, these indices, except the PLR and NLPR, were significantly lower in the BG than in the TG. However, none of these HIIs showed significant differences between the PG and TG. Consequently, baseline complete blood count-derived HIIs may be considered in differentiation between *Brucella* and other infectious types of spondylodiscitis. However, these HIIs may not be used in the discrimination of pyogenic from tuberculous spondylodiscitis. Since this is the first study on the topic, its results should be confirmed by future studies.

Given that the methods used for the diagnosis and differential diagnosis of infectious spondylodiscitis are laborious, time-consuming, costly, impractical, and difficult to access, it is understood that more appropriate assessment approaches are needed. In this respect, the calculation of HIIs to differentiate between *Brucella*, pyogenic, and tuberculous spondylodiscitis provides a simple and cost-effective approach. In addition, it is well known that there are some differences between different infectious diseases in terms of blood cells. For example, unlike many other infections, *Brucella* infection is associated with a reduction in the number of blood cells [30], which are used in the calculation of HIIs. Therefore, a significant role of HIIs in distinguishing between different pathogens responsible for infectious diseases can be expected. In this regard, the present study has a scientific and logical basis.

Another important point to mention is that the patients’ baseline (pre-treatment) data were used in the present study. As the pre- and post-treatment complete blood count are different, the baseline (pre-treatment) data should be used to assess their performance in diagnostic and differential procedures. For example, while the baseline (pre-treatment) data for brucellosis may show cytopenia, an increase in the number of blood cells may be observed during the treatment period due to the effects of the therapeutic agents used, such as antibiotics and steroids [31]. Accordingly, baseline data were used in this study as it focused on the early differential diagnosis of microorganisms responsible for infectious spondylodiscitis. In this way, the results of the current study were free from the effects of factors such as the late stage or treatment of the diseases discussed and were able to reach the early changes necessary for early diagnosis and differential diagnosis.

The present study showed that patients with *Brucella* spondylodiscitis had lower HII scores than those with pyogenic spondylodiscitis. Therefore, although an increase in HII scores generally indicates infection-related conditions such as inflammation and immune response [32], a decrease in HII scores indicates *Brucella* infection-related conditions such as *Brucella* spondylodiscitis. This was a logically expected finding given that HIIs are complete blood count-derived indicators and reduced blood cells are associated with *Brucella* infection [30]. In contrast with the present findings, a recent study reported that brucellosis was associated with high HII scores [33]. This incompatibility may be related to complete blood count performed at different stages of brucellosis.

This study also showed that patients with *Brucella* spondylodiscitis had lower HII scores, except for the PLR and NLPR, than those with tuberculous spondylodiscitis. The similarity between *Brucella* and tuberculous spondylodiscitis in terms of the PLR and NLPR may be related to lymphopenia [34] and thrombocytopenia [35] secondary to tuberculosis. This is because lymphocyte and platelet counts are used in the calculation of these indices, and lymphopenia and thrombocytopenia can be seen in tuberculosis infection [34,35].

Although the present study was primarily concerned with complete blood count-derived HIIs to differentiate between infectious spondylodiscitis types, patients with tuberculous spondylodiscitis had a significantly longer symptom duration than those with *Brucella* or pyogenic spondylodiscitis. Therefore, this finding suggests that comparisons based on the symptom duration can be used in the differential diagnosis of infectious types of spondylodiscitis.

Given the challenges in the diagnosis, differential diagnosis, and causative pathogen detection of infectious spondylodiscitis, a comparative analysis including a combination of clinical, laboratory, and radiological findings is required for early and effective management [36,37,38]. In this context, HII analyses performed either alone or in combination with other scores may be useful in the early and effective management of infectious spondylodiscitis. Therefore, the potential contribution of HIIs as an adjunct to previous assessments including clinical, laboratory, and radiological findings should be considered in future studies. Another potential contribution of HIIs could be tests and methods to distinguish between infectious and non-infectious types of spondylodiscitis, which is also worth investigating for future studies.

There are a limited number of studies in the literature that focus on the comparative analysis of *Brucella*, pyogenic, and tuberculous spondylodiscitis [39,40,41]. Moreover, to our knowledge, no comparative study addresses the HIIs in these types of infectious spondylodiscitis. In view of the challenges in diagnostic, differentiative, and therapeutic processes leading to severe complications, the necessity of more effective management becomes apparent [3,5,6,8]. Thus, researchers have attempted to improve the outcomes of infectious spondylodiscitis and focused on faster diagnostic, differentiative, and therapeutic processes [13,16,17,18,19]. Baseline differences between the types of infectious spondylodiscitis may yield an early diagnosis and provide the application of the most relevant antibiotics. The present study was planned and conducted with this positive point of view and vision.

Previous studies have addressed the differences between infectious spondylodiscitis types in terms of their demographic, clinical, radiological, and laboratory features [38,39,40,41,42,43,44,45]. Although complete blood count-derived HIIs have been evaluated in various medical conditions considering their diagnostic [23,24], prognostic [21,25,46], predictive [22,26,47,48], and treatment response [27] ability, no study to date has addressed the role of these indices in the differentiation between *Brucella*, pyogenic, and tuberculous spondylodiscitis. Therefore, we cannot present comparative results from different studies.

On the other hand, the present study has some limitations that should be acknowledged. Firstly, its retrospective design may have caused missing data, including symptoms (e.g., fever, pain, weight loss, and loss of appetite) and microbiological and host features (e.g., bacterial resistances, immunity, and comorbidity). This may have biased the results of this study. Both possible missing data and the lack of a healthy control group in the study may lead to a partial reduction in the reliability of the results. Secondly, it was performed at a single center and included a small sample size. Thirdly, its results may not be generalizable to other populations where brucellosis is not endemic. Also, as the microbiological agents of spondylodiscitis vary regionally [49], the region should be considered when evaluating the present results. Fourthly, it mainly focused on baseline complete blood count-derived HIIs in the differential diagnosis of infectious spondylodiscitis types and did not consider their potential roles in prognosis, prediction, and treatment monitoring. To our knowledge, no study is present on the topic; an in-depth analysis and discussion could not be performed, and further studies are needed.

In conclusion, the management of infectious spondylodiscitis has some challenges. Due to non-specific clinical features and various difficulties in diagnosis, a delay in differentiation between responsible microorganisms may occur. Thus, severe complications may develop in patients with infectious spondylodiscitis, when difficulties arise in diagnostic, differentiative, and therapeutic processes. To prevent this, it may be useful to consider the initial distinguishing features between infectious spondylodiscitis types. With this reasoning, we conducted the current study. As a result, patients with brucella spondylodiscitis have lower NLR, MLR, PLR, NLPR, SII, SIRI, and AISI scores than those with pyogenic spondylodiscitis. Also, patients with brucella spondylodiscitis have lower NLR, MLR, SII, SIRI, and AISI scores than those with tuberculous spondylodiscitis. These findings may be considered in the differentiation between *Brucella* spondylodiscitis and other infectious spondylodiscitis types. However, pyogenic spondylodiscitis and tuberculous spondylodiscitis are similar in terms of these indices.

## Figures and Tables

**Table 1:** Complete blood count-derived hematological inflammatory indices.

Indices	Derivation
NLR	Neutrophil/Lymphocyte
MLR	Monocyte/Lymphocyte
PLR	Platelet/Lymphocyte
NLPR	Neutrophil/(Lymphocyte × Platelet)
SII	Neutrophil × Platelet/Lymphocyte
SIRI	Neutrophil × Monocyte/Lymphocyte
AISI	Neutrophil × Platelet × Monocyte/Lymphocyte

SII: systemic immune/inflammation index; SIRI: systemic inflammatory response index; AISI: aggregate index of systemic inflammation.

**Table 2:** Baseline values in *Brucella*, pyogenic, and tuberculous groups.

	*Brucella* Spondylodiscitis (n = 51)	Pyogenic Spondylodiscitis (n = 43)	Tuberculous Spondylodiscitis (n = 22)	*p*
Age, years	49.1 ± 17.2 (18–79)	53.4 ± 15.2 (19–84)	45.6 ± 19.4 (18–82)	0.669 ^a^ 1.0 ^b^ 0.245 ^c^
Gender (F/M)	20/31	14/29	10/12	0.526 ^a^ 0.796 ^b^ 0.416 ^c^
Symptom duration, days	52.5 ± 38.9 (10–200)	73.7 ± 48.5 (7–240)	195.7 ± 192.4 (30–730)	0.804 ^a^ <0.001 ^b^ <0.001 ^c^
CRP, mg/L	39.9 ± 31.6 (3–136)	66.8 ± 55.1 (3–193)	51.6 ± 59.0 (3–242)	0.021 ^a^ 1.0 ^b^ 0.662 ^c^
ESR, mm/h	39.0 ± 21.4 (2–93)	53.1 ± 27.0 (3–140)	44.5 ± 25.5 (13–101)	0.019 ^a^ 1.0 ^b^ 0.548 ^c^

The data were given as mean ± standard deviation (min.–max.) or number. CRP: C-reactive protein, ESR: erythrocyte sedimentation rate, ^a^: comparison between *Brucella* and pyogenic groups; ^b^: comparison between *Brucella* and tuberculous groups; ^c^: comparison between pyogenic and tuberculous groups.

**Table 3:** Baseline complete blood count values in *Brucella*, pyogenic, and tuberculous groups.

	*Brucella* Spondylodiscitis (n = 51)	Pyogenic Spondylodiscitis (n = 43)	Tuberculous Spondylodiscitis (n = 22)	*p*
Leukocyte count (10^9^/L)	6.7 ± 2.1 (3.4–12.8)	10.2 ± 3.8 (2.4–24.8)	8.6 ± 3.0 (4.9–16.4)	<0.001 ^a^ 0.044 ^b^ 0.147 ^c^
Neutrophil count (10^9^/L)	4.0 ± 1.8 (1.2–8.6)	7.5 ± 3.8 (1.5–22.3)	6.0 ± 2.8 (2.7–13.8)	<0.001 ^a^ 0.019 ^b^ 0.139 ^c^
Lymphocyte count (10^9^/L)	2.0 ± 0.6 (1.1–3.5)	1.8 ± 0.8 (0.6–3.8)	1.8 ± 0.7 (0.8–3.5)	0.883 ^a^ 0.544 ^b^ 1.0 ^c^
Monocyte count (10^9^/L)	0.6 ± 0.3 (0.1–1.6)	0.6 ± 0.3 (0.2–1.4)	0.7 ± 0.3 (0.3–1.5)	0.599 ^a^ 0.362 ^b^ 1.0 ^c^
Platelet count (10^9^/L)	263.4 ± 72.6 (137.0–423.0)	305.1 ± 109.4 (84.0–588.0)	297.4 ± 62.6 (206.0–431.0)	0.065 ^a^ 0.380 ^b^ 1.0 ^c^

The data were given as mean ± standard deviation (min.–max.). ^a^: Comparison between *Brucella* and pyogenic groups; ^b^: comparison between *Brucella* and tuberculous groups; ^c^: comparison between pyogenic and tuberculous groups.

**Table 4:** Baseline complete blood count-derived indices in *Brucella*, pyogenic, and tuberculous groups.

	*Brucella* Spondylodiscitis (n = 51)	Pyogenic Spondylodiscitis (n = 43)	Tuberculous Spondylodiscitis (n = 22)	*P*
NLR	2.12 ± 1.20 (0.7–5.6)	4.84 ± 3.52 (1.0–14.1)	4.0 ± 2.89 (1.5–12.6)	<0.001 ^a^ 0.007 ^b^ 0.280 ^c^
MLR	0.28 ± 0.12 (0.1–0.6)	0.40 ± 0.28 (0.1–1.7)	0.45 ± 0.36 (0.1–1.5)	0.027 ^a^ 0.038 ^b^ 0.792 ^c^
PLR	141.2 ± 58.4 (59.1–310.9)	196.9 ± 149.5 (64.6–920.0)	196.7 ± 110.0 (86.0–538.7)	0.048 ^a^ 0.151 ^b^ 1.0 ^c^
NLPR	0.008 ± 0.005 (0.0–0.2)	0.017 ± 0.015 (0.0–0.7)	0.013 ± 0.009 (0.1–0.3)	<0.001 ^a^ 0.173 ^b^ 0.413 ^c^
SII	576.3 ± 389.4 (112.0–1586.1)	1537.6 ± 1426.4 (180.9–7728.0)	1250.0 ± 1109.5 (298.2–5090.4)	<0.001 ^a^ 0.034 ^b^ 0.861 ^c^
SIRI	1.23 ± 1.03 (0.2–4.4)	3.27 ± 3.22 (0.4–14.1)	3.11 ± 3.68 (0.5–14.1)	<0.001 ^a^ 0.001 ^b^ 0.570 ^c^
AISI	333.0 ± 301.3 (44.8–1192.5)	1063.2 ± 1307.3 (36.2–7728.0)	991.5 ± 1308.8 (134.9–5090.4)	<0.001 ^a^ 0.001 ^b^ 0.589 ^c^

The data were given as mean ± standard deviation (min.–max.). NLR: neutrophil/lymphocyte; MLR: monocyte/lymphocyte; PLR: platelet/lymphocyte; NLPR: neutrophil/(lymphocyte × platelet); SII: immune inflammatory index; SIRI: systemic inflammatory response index; AISI: aggregate index of systemic inflammation. ^a^: Comparison between *Brucella* and pyogenic groups; ^b^: comparison between *Brucella* and tuberculous groups; ^c^: comparison between pyogenic and tuberculous groups.

## Data Availability

The datasets on patient material are not publicly available as they also contain individual participants’ details. These data can be obtained from the corresponding author in an anonymized form upon reasonable request.
